# Pharmacological options for Candida albicans Endocarditis at the roadblock with irrecoverable prosthetics and drug interactions: a case report and review of literature

**DOI:** 10.1186/s12879-023-08267-z

**Published:** 2023-05-08

**Authors:** Syeda Sahra, Aneeqa Javed, Abdullah Jahangir, Sharanjeet K. Thind

**Affiliations:** 1 Department of Infectious Diseases, Veterans Affairs Medical Center, Oklahoma City, OK 73104 USA; 2 Department of Infectious Diseases, The University of Oklahoma Health Sciences Center (OUHSC), Oklahoma City, OK 73104 USA; 3 Department of Internal Medicine, Staten Island University Hospital, Staten Island, NY 10305 USA; 4 Department of Critical Care, Veterans Affairs Medical Center, Oklahoma City, OK 73104 USA

**Keywords:** *Candida albicans*, Endocarditis, Fungemia, Candidemia, Cardiac devices, Prosthetic valves

## Abstract

**Background:**

Candidemia is the fourth most common nosocomial bloodstream infection. Endocarditis from candidemia is a rare but possibly fatal complication. The efficacy of amphotericin and echinocandins for induction and azoles for suppression has been well studied. Source control of infection, including removal of foreign bodies, remains the cornerstone for the success of any antifungal therapy.

**Case Presentation:**

We are describing a case of a 63-years old patient with multiple comorbidities who developed candidemia secondary to *Candida albicans*. The prospect of curing the fungemia was made difficult by prosthetic devices, including prosthetic heart valves, intracardiac defibrillator, and inferior vena filter, which could not be extracted due to poor cardiovascular status and higher postoperative mortality risk. Combination therapy with amphotericin and 5-Flucytosine (5FC) was used with the first recurrence. Suppression with fluconazole was contraindicated due to prolonged corrected QT (QTc) interval. Isavuconazole was employed for chronic lifelong suppression.

**Conclusion:**

Retaining prosthetics in higher surgical risk patients presents us with unique clinical and pharmacological challenges regarding breakthrough infections, drug interaction, and side effects from prolonged suppressive therapies.

**Supplementary Information:**

The online version contains supplementary material available at 10.1186/s12879-023-08267-z.

## Background

Candidemia is the fourth most common healthcare acquired bloodstream infection [1]. An analysis of 128 cases presenting to a teaching hospital in London with candidemia found serious complications such as endocarditis and endophthalmitis in 6% of the cases [2]. Candida endocarditis is a rare disease occurring frequently in patients with prosthetic cardiac valves and devices, with a mortality rate described to be as high as 40% [3]. *Candida albicans* are the most frequently isolated *Candida* species from blood cultures. Patients are usually treated with amphotericin B and azoles, along with surgical valve replacement or device removal, if any [4]. Device and catheter removal is highly encouraged when feasible, as *Candida* is known for making biofilms on foreign bodies. Halawa et al. (2011) reviewed the association and increased incidence of *Candida* endocarditis with cardiac rhythm management devices. They described amphotericin B with or without 5-Flucytosine (5FC) or an echinocandin such as micafungin as primary therapy and device removal [5]. Since there is a risk of relapse without surgical therapy, some patients are given oral azoles for chronic suppression [6, 7]. However, complications may arise when treating these patients, such as multidrug resistance organism infection, comorbidities, and drug-drug interactions [8].

Case Presentation:

We are describing a case of a 63-year-old male with a history of coronary artery disease status, heart failure with low ejection fraction (EF), and lower extremity deep venous thrombosis (DVT). His surgical history was significant for coronary artery bypass graft surgery, prosthetic bovine mitral and tricuspid valves placement, intracardiac defibrillator (ICD) placement (two years ago), and an inferior vena cava (IVC) filter placed six months ago. He was admitted to the hospital with weakness and low-grade fever. Initial workup was significant for yeast in blood cultures which was identified as *Candida albicans*. He was treated with intravenous (IV) micafungin, and blood cultures were cleared of *Candida* after five days. The fundoscopy evaluation was done, and no ophthalmology involvement was seen. Transesophageal echocardiogram (TEE) revealed perforated mitral and tricuspid prosthetic valves with mobile vegetations along both surfaces of valves. The EF was noted to be less than 10% (Video-1). The risks and benefits of removing all prosthetics, including prosthetic cardiac valves, IVC filter, and ICD to treat fungemia, were explicated to the patient. However, given the high intraoperative risk due to low EF, the patient declined surgical removal of the prosthesis and opted to be treated with antifungals. Fluconazole could not be used due to his prolonged QTC at baseline (> 550 ms) and increased risk of bleeding with the anticoagulants (drug-drug interaction with apixaban). The patient was treated with six weeks of IV micafungin followed by lifelong suppressive therapy planned with oral isavuconazole. Two months later, the patient presented to the hospital with weakness, and his blood cultures were positive again for *Candida albicans*. The clinical course in this second hospitalization was complicated by a transient ischemic attack and left lower leg ischemia needing an above-the-knee amputation. Both vascular events were deemed secondary to fungal emboli based on the operative report and cultures. The fungemia was persistent, and amphotericin with flucytosine was given for seven days. The antifungals were then switched to micafungin again once the cultures cleared after eight days. The surgical source control could not be pursued in respect of his wishes, and he was discharged with oral suppressive isavuconazole. The timeline with choice of antifungals has been described in Table-1. The patient was reported to be doing well on one month follow-up.

## Discussion

A recent study by Meena et al. (2022), which retrospectively reviewed the literature for 250 cases of fungal endocarditis, highlighted improved survival outcomes in patients with surgical intervention (Hazard ratio 0.20, 95% confidence interval 0.09–0.42; p-value < 0.001) [3]. Improvement in patient survival parameters and clearance of blood cultures after extraction of cardiac implant electronic devices was reported in a retrospective study by Baman et al. [9]. While the statistics reiterate the brighter outcomes with valve replacements and device extractions, real-life clinical cases get complicated. Issues including but not limited to poor surgical candidacy and concurrent debilitating comorbidities, older age, recurrent endocarditis, active intravenous drug abuse, and patient directives are one of the common grounds for not pursuing surgery in patients with fungal endocarditis.

The efficacy of amphotericin B for induction has been established and reinforced most recently by the results of the ESCAPE trial, where patients with amphotericin B had better survival outcomes compared to the subgroup where echinocandin was used for induction [10]. A retrospective study by Fioriti et al. (2022) reviewed combination therapies, including clinical and in vitro studies. While outcomes from the echinocandin plus azoles combination and polyenes plus azoles combination were highly variable, hopeful results were seen with the combination of amphotericin B and 5FC. A reciprocal potentiation was also observed in other *Candida* species, which are historically deemed difficult to treat (including *C. auris, C. glabrata *and *C. krusei*) [11]. Amphotericin, a polyene, is a potent fungicidal drug that primarily targets the membranous ergosterol, whereas 5FC, a pyrimidine, acts synergistically by inhibiting protein synthesis. These two unrelated mechanisms of action can explain the synergistic advantage. Following these anecdotes in the literature, after the first breakthrough infection, we opted to add 5FC to effectively lower the infection burden before transitioning to the suppressive regimen, which aligned with his goals of care. The duration of induction therapy with amphotericin and 5FC remains to be determined in cases where the source control is not established. Amphotericin is a potent nephrotoxic drug, and while 5FC usually causes adverse gastrointestinal symptoms, there is a risk of bone marrow suppression with prolonged use. Both drugs cannot be used in ambulatory settings like echinocandins due to the need for persistent monitoring of cell counts, electrolytes, and renal function.

Current guidelines for oral suppressive therapies are based mainly on experience with fluconazole. A meta-analysis reported by Ahmed et al. (2011) has described a promising 95% cure rate of endocarditis in patients who received fluconazole as chronic suppressive therapy [12]. But fluconazole still might not work in patients who cannot tolerate it due to prolonged QTC and drug interactions. In our patient with a prolonged QTc interval, fluconazole was avoided. Fluconazole is a CYP3A4 inhibitor and might increase the serum concentration of apixaban. Increased risk of hospitalization secondary to bleeding [OR 3.5 (1.4–10.6), 95% confidence interval] has been described in a case-control study with more than 32,000 patients [13]. Similarly, a 2.35-fold increased incidence of major bleeding was reported with the use of fluconazole in patients who were either taking apixaban or another non-vitamin K antagonist (either rivaroxaban or dabigatran) in a study published in JAMA in 2017 by Chang SH et al. [14]. This data left us to pick an alternative oral antifungal for secondary prophylaxis/a suppressive treatment regimen or another oral anticoagulant. Fluconazole increases the serum concentration of vitamin K antagonists and would need frequent international normalized ratio (INR) monitoring. The patients’ compliance with routine bloodwork from past could have been better. The option of continuing subcutaneous enoxaparin at home, which was well tolerated during hospital stays, was also discussed with the patient, but the idea of injecting himself every day was different from his goals of care.

The non-inferiority of isavuconazole compared to caspofungin for candidemia and invasive infections was established in the ACTIVE trial [12]. So, we used isavuconazole which is highly active against *Candida* species, particularly *Candida albicans* [14]. It has also been shown to shorten the QT interval [15].

We are reporting this case as we faced a disease control dilemma in cases where foreign bodies with fungal infections cannot be taken out. Our patient, with retained prosthetics, had a breakthrough infection after being treated appropriately with IV micafungin and while on isavuconazole prophylaxis and achieved culture clearance sooner the second time when both amphotericin and flucytosine were employed. Despite the clinical trials done to assess the efficacy of single versus double antifungal therapies in persistent fungemia, there needs to be more data for effective treatment and suppressive regimens in cases where the surgical source control is hard to be established. Future clinical trials with the subgroups with retained prosthetic valves and devices can provide more guidance.

**Table 1:** Timeline of the antifungal therapies received during hospitalization

Timeline of Treatment	Antifungal used	Route	Dosage	Frequency	Duration
Day 1 – Day 45	Micafungin	Intravenous	100 mg	Daily	45 days
Day 46–47	Isavuconazole	Oral	372 mg	Every 8 h	2 days
Day 48–107	Isavuconazole	Oral	372 mg	Daily	60 days
Day 108–115	Amphotericin plus Flucytosine	Intravenous	Amphotericin: 5 mg/kg Flucytosine: 50 mg/kg	Daily	7 days
Day 116–125	Micafungin	Intravenous	100 mg	Daily	9 days
Day 126-present	Isavuconazole	Oral	372 mg	Daily	Ongoing.

## Electronic supplementary material

Below is the link to the electronic supplementary material.

Supplementary Material 1 Video 1: Video clip from a transesophageal echocardiogram (TEE) showing backflow, supporting the low ejection fraction. perforated mitral and tricuspid prosthetic valves with mobile vegetations along both surfaces of valves.

## Data Availability

The hospital course information and literature datasets used or analyzed during the current case reports are available from the corresponding author upon reasonable request.
